# Mothers’ Experience With Health Insurance Coverage When Youngest Child Reaches 18 Years of Age

**DOI:** 10.1001/jamahealthforum.2022.5514

**Published:** 2023-02-17

**Authors:** Eric Napierala, Sashoy Patterson, Ana Laboy, Mark Weiss, Jessica Smith

**Affiliations:** 1 Georgia Health Policy Center, Andrew Young School of Policy Studies, Georgia State University, Atlanta; 2 Department of Political Science, College of Arts and Sciences, Emory University, Atlanta, Georgia

## Abstract

This cross-sectional study uses data from the American Community Survey to track maternal insurance coverage status as children age from infancy to adulthood.

## Introduction

The Affordable Care Act has provided health insurance coverage to millions of individuals in the US since its enactment in 2010.^1^ Nevertheless, a substantial portion of the population continues to be uninsured.

Recent research has focused on insurance rates during the postpartum period for mothers and the association with maternal morbidity and mortality^2^; however, policies that contribute to postpartum coverage cliffs (eg, Medicaid eligibility limits) can also affect mothers at other stages in life.^3,4,5^ Using data from the American Community Survey (ACS), the present analysis tracks maternal insurance coverage status as children age from infancy to adulthood. We find that uninsurance rates increase for mothers at 2 points in their child’s life: (1) when the child is younger than 1 year and (2) when the child turns 18 years old.

## Methods

The US Census Bureau’s ACS microdata were collected through the Integrated Public Use Microdata Series USA data portal for the first 5 years of Medicaid expansion, from 2014 through 2018. The sample used in this study includes all female adolescents and adults aged 15 to 64 years who reported the age of their youngest child still living in the household with state and total family income as a percentage of the federal poverty threshold. State Medicaid expansion status was collected from the Kaiser Family Foundation, and a dummy variable was created for states that expanded Medicaid during the survey year. The ACS’s person weightings were used to estimate the total number of people within each stratification.

Self-reported types of health insurance coverage included employer provided, privately purchased, Medicaid, Medicare, TRICARE, or Veterans Administration provided. Individuals without those types of coverage were considered uninsured (including those with Indian Health Services–provided coverage, as these plans are not always comprehensive). This study followed STROBE reporting guidelines, and the institutional review board at Georgia State University waived approval because data sets used in the study were designated not human participant research.

## Results

The Figure illustrates the uninsurance rate for mothers by age of youngest child and state’s Medicaid expansion status. While mothers had relatively higher uninsurance rates before their youngest child was of school age (around 5 years old), the rate steadily decreased until the child was 17 years old. When the child turned 18 years old, these rates then increased 2.7 percentage points in nonexpansion states and 1.1 percentage points in expansion states.

**Figure.**
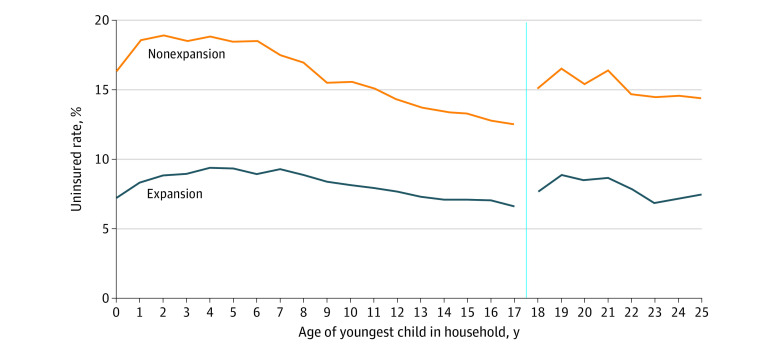
Uninsurance Rates Among Mothers by State Medicaid Expansion Status and Age of Youngest Child in the Household The blue vertical line indicates the point of parental Medicaid eligibility loss due to the youngest dependent turning 18 years old.

The Table summarizes the study sample by state expansion status and level of family income relative to the poverty threshold for Medicaid eligibility under the Affordable Care Act. The uninsured rate increased among all income groups when the youngest child living in the household turned 18 years old, regardless of state expansion status. The largest uninsurance rate increases occurred in nonexpansion states, with uninsurance in the lowest income group jumping 5.8 percentage points. Expansion states also saw a rise, but the lowest income group experienced the smallest increase, while uninsurance in the group at 139% to 200% of the federal poverty level (FPL) increased 2.9 percentage points.

**Table.** Uninsurance Rates Among Mothers by State Medicaid Expansion Status and Poverty Group^a^

Federal poverty level	Expansion states, %			Nonexpansion states, %		
	Child 17 y old	Child 18 y old	Difference between 18 and 17 y old	Child 17 y old	Child 18 y old	Difference between 18 and 17 y old
>200%	4.7	5.7	0.9	7.8	9.7	2.0
139%-200%	13.5	16.4	2.9	23.6	25.8	2.1
≤138%	14.8	15.3	0.5	30.2	36.0	5.8

^a^ Based on authors’ analysis of data from the Integrated Public Use Microdata Series USA database for the first 5 years of Medicaid expansion, from 2014 through 2018.

## Discussion

This cross-sectional study demonstrated that a potential driver of higher uninsurance rates, particularly among lower-income households, was the loss of parental Medicaid eligibility when the youngest dependent turned 18 years old.^6^ Expansion states allow parents of adult children to remain eligible for Medicaid when earning up to 138% of the FPL, while the same individuals lose coverage in nonexpansion states. Additionally, households earning less than 100% of the FPL in nonexpansion states are not eligible to receive individual marketplace coverage subsidies.

The data set used in this study has 3 notable limitations. First, ACS surveys are self-reported and therefore are not as accurate as official records. Second, ACS surveys are conducted monthly but reported yearly; consequently, we could not determine if a survey was taken before or after a state expanded Medicaid. Third, the variable detailing the youngest child’s age depended on whether the child still lived at home. Nonetheless, we demonstrate that differing Medicaid policies showed contrasting outcomes for individuals—expansion states may provide a safety net, while nonexpansion states may exacerbate the uninsurance rate due to stricter eligibility criteria.

## Notes

Supplement. Data Sharing Statement
