# Supplementary material for: Mothers’ Experience With Health Insurance Coverage When Youngest Child Reaches 18 Years of Age
Source: JAMA Health Forum. 2023 Feb 17;4(2):e225514. doi: 10.1001/jamahealthforum.2022.5514 (PMC9938430; doi:10.1001/jamahealthforum.2022.5514)
Supplement: Supplement. — Data Sharing Statement [file jamahealthforum-e225514-s001.pdf]

Data Sharing Statement

Napierala. Mothers' Experience With Health Insurance Coverage When Youngest Child Reaches 18 Years of Age. *JAMA Health Forum*. Published February 17, 2023. doi:10.1001/jamahealthforum.2022.5514

Data
Data available: Yes
Data types: Deidentified participant data
How to access data: Data can be pulled from https://usa.ipums.org/usa/ or email enapierala1@gsu.edu for access to files used in study.
When available: With publication

Supporting Documents
Document types: None

Additional Information
Who can access the data: Anyone requesting the data.
Types of analyses: Any purpose.
Mechanisms of data availability: Without investigator support.
Any additional restrictions: None.
